# Finding self-worth—Experiences during a multimodal rehabilitation program when living at a residency away from home

**DOI:** 10.1080/24740527.2020.1810001

**Published:** 2020-10-05

**Authors:** Linda Spinord, Ann-Charlotte Kassberg, Britt-Marie Stålnacke, Gunilla Stenberg

**Affiliations:** a Department of Community Medicine, Umeå University, Umeå, Sweden; b Department of Development and Research, Region Norrbotten, Luleå, Sweden; c Department of Health Science, Luleå University of Technology, Luleå, Sweden

**Keywords:** chronic pain, MMRP, grounded theory, group rehabilitation, interview

## Abstract

**Background:**

Patients with chronic pain who live in rural areas often need to travel long distances to participate in multimodal rehabilitation programs. To reduce traveling during the programs, patients sometimes live at a residency close to the clinic and thus far from home.

**Aims:**

The aim of this study was to explore how patients with chronic pain experience participation in an multimodal rehabilitation program while living at a residency.

**Method:**

Twelve patients from two specialist clinics in northern Sweden were interviewed about their experiences of participating in a multimodal rehabilitation program. The data were analyzed qualitatively using a grounded theory method with an emergent design.

**Results:**

The analyses resulted in a model with the core category “finding my self-worth” consisting of four categories: “space for myself,” “mirroring myself,” “I am of value,” and “dealing with returning to everyday life.” The model illustrates the process whereby participants are given space for themselves and an opportunity to mirror themselves in interaction with other participants. That provided insight about their self-worth that was valuable for return to everyday life at home and work.

**Conclusion:**

Living at a residency during multimodal rehabilitation provided added value when patients were relived from the obligations of everyday life at home and given time for reflection and interaction with others in similar situations. This contributed to awareness of their own value and the necessity of taking care of themselves. This new insight led to increased motivation to act differently at home.

## RÉSUMÉ

**Contexte:** Les patients souffrant de douleur chronique qui vivent en milieu rural doivent souvent parcourir de longues distances pour participer à des programmes de réhabilitation multimodale. Pour réduire les déplacements pendant les programmes, les patients vivent parfois dans une résidence à proximité de la clinique et donc loin de chez eux.

**Objectifs:** Le but de cette étude était de se pencher sur la façon dont les patients souffrant de douleur chronique vivent leur participation à un programme de réadaptation multimodale lorsqu’ils vivent en résidence.

**Méthode:** Douze patients provenant de deux cliniques spécialisées du Nord de la Suède ont été interviewés sur leur participation à un programme de réadaptation multimodale. Les données ont été analysées de manière qualitative en utilisant la méthode de la théorie ancrée et un devis émergent.

**Résultats:** Les analyses ont donné lieu à un modèle dont la catégorie principale, « trouver ma valeur personnelle », consiste en quatre catégories : « un espace pour moi », « mon reflet », « j’ai de la valeur » et « le retour à la vie de tous les jours ». Ce modèle illustre le processus par lequel les participants disposent d’un espace pour eux-mêmes et ont l’occasion de voir leur propre reflet, en interaction avec les autres participants. Cela leur a permis de mieux percevoir leur propre valeur personnelle, ce qui a été d’une grande utilité pour leur retour à la vie quotidienne à la maison et au travail.

**Conclusion:** Le fait de vivre en résidence pendant une réadaptation multimodale a apporté une valeur ajoutée lorsque les patients ont été libérés des obligations de la vie quotidienne à la maison et ont eu le temps de réfléchir et d’interagir avec d’autres personnes dans des situations similaires. Cela a contribué à la prise de conscience de leur propre valeur et de la nécessité de prendre soin d’eux-mêmes. Cette nouvelle perspective a conduit à une motivation accrue pour agir différemment à la maison.

## Introduction

Chronic pain is often accompanied by sleep disturbance, fatigue, cognitive dysfunctions, emotional distress, fear of movement, and catastrophizing.^1^ Such health-related difficulties can lead to an inability to pursue a career or education, as well as limited possibilities to engage in social life and leisure activities.^2–4^ Additionally, because pain is unpredictable and may vary from day to day, evidence indicates that people with chronic pain may find it difficult to establish and maintain emotional and physical contacts with other persons.^2,4^ Thus, due to an ability to perform their previous range of roles and social behaviors, persons living with chronic pain often need to make numerous lifestyle changes.^5^

A multimodal rehabilitation program (MMRP) is an established option for the treatment of chronic pain.^6,7^ These programs are provided to patients whose regular pain treatment, such as medicine or a self-training program, has been insufficient. The aim of MMRP is to reduce the risk of health-related complications by improving a person’s ability to cope with the consequences of pain, to increase participation in society, and to facilitate return to work.^4^ Systematic reviews have shown that MMRPs are more effective than single treatments.^4,6,7^ MMRP is a method developed to treat patients with moderate and complex rehabilitation needs. The method is based on a biopsychosocial model with a focus on biological, psychological, and social factors. It is a logical team-based cognitive–behavioral therapy program that combines physiological, pedagogical, and physical interventions such as activities and exercise.^4,8^ However, a recent qualitative systematic review revealed difficulties in maintaining self-management learned from participation in an MMRP when experiencing chronic pain and that motivation sometimes diminishes over time. Additionally, support groups and booster sessions were shown to be important in sustaining new strategies.^9^ Previous qualitative studies^10,11^ have developed models for understanding patients’ experiences of participation in an MMRP. These models describe patients’ experiences of an ongoing change process from chaos and despair to acceptance and improvements in self-image as well as life and work roles. Furthermore, a continuous exchange of emotions, thoughts, and knowledge was perceived as being effective.

The prevalence of moderate to severe chronic pain in Sweden has been estimated to be 18%.^1^ MMRPs are available throughout Sweden, but their design and content can differ significantly, depending on geographical and organizational factors in the health care system.^12^ In northern Sweden, with its geographically dispersed and sparsely populated areas, MMRPs in specialist care are provided at a county hospital or a university hospital. In a recent study from northern Sweden, it was shown that patients improved regardless of the different designs.^13^ However, the geographical conditions often result in long distances between the specialist clinics and the populations they serve in rural areas. This can be troublesome for patients with chronic pain due to difficulties due to pain that affect their ability to travel by public or private transportation for an extended amount of time. Additionally, public transport in the sparsely populated areas of Sweden is not always adapted for persons with disabilities. Consequently, participants in MMRPs may require accommodation in closer proximity to the clinic at which the program takes place. In northern Sweden, patients are often able to stay at a designated hotel that provides specialist care and thus can live away from home for several weeks. This situation differs from other parts of Sweden where living at a residency during an MMRP is not a customary praxis.

A previous review investigated the experiences and economic factors of living at a residency during treatment in comparison to traditional hospital accommodation.^14^ The authors concluded that patients experienced greater freedom, privacy, and independence at a residency, which was also a cost-effective alternative to traditional hospital accommodation. Moreover, another study^15^ investigated the experiences of patients with breast cancer who were living at a residency during radiotherapy treatment. Patients reported advantages of staying at a residency, such as feeling safe and the possibility to resume a new everyday life, but also disadvantages such as intruding self-image and an increased vulnerability during the stay.

Because living at a residency may have an impact on an individual’s rehabilitation period, it is of importance to explore patients’ experiences of a residency stay concurrent with participation in an MMRP. To our knowledge, no studies have explored this before. The aim of this study was to explore how patients with chronic pain experience participation in an MMRP while living at a residency.

## Methods

### Design

This study used a modified constructivist grounded theory approach to conduct and analyze interviews and construct a theory according to Charmaz.^16^ This approach was applied with the aim of producing a theoretical understanding of the social processes and relations involved when the study participants lived at a residency while participating in an MMRP. Grounded theory was chosen because it focuses on uncovering patients’ perspectives and processes, thereby making it possible to comprehend the underlying patterns.^16^

### Settings

The study was conducted in two specialist clinics in two county councils in northern Sweden. The clinics were the main centers for pain rehabilitation with outpatient programs in the regions, which were largely rural areas with a low population density.

The residency was located near the respective rehabilitation clinics, and patients paid only a small fee for accommodation including housing and full board. Assistive personnel at the residency prepared breakfast and meals, booked transports, and, if needed, referred patients to professionals on the team. At the residency, several indoor and outdoor arenas are available where the patients can perform activities alone or with others: a room with Internet connection, crafting studios, a training room, and a relaxation facility with a sauna. Activities at the residency were organized and facilitated by professionals but could also be performed on a voluntary basis by the patients themselves. In the hotel lobby, there is an open space and social area where the patients can meet others (patients and/or professionals), read newspapers, or simply socialize with other people between participating in rehabilitation interventions or mealtimes.

During the MMRP, the patients created a rehabilitation plan together with the team and were encouraged to take an active role in goal setting. The majority of the interventions were conducted in group sessions lasting approximately 6 h/day for 3 or 4 weeks, based on cognitive–behavioral principles. The MMRP included exercise, body awareness, activity training, ergonomic practice, occupational strategies, and information about bodily and psychological reactions to chronic pain. The participants underwent individually tailored sessions with the team members. The general goals of the programs were to improve activity levels and life satisfaction, as well as to improve the participants’ coping strategies to allow them to achieve their individual goals. For more details about the programs, the reader is referred to a previous study.^13^

### Participants

The following inclusion criteria were used for participation in the study: (1) age between 18 and 65 years, (2) completion of an MMRP in a specialist pain rehabilitation clinic in northern Sweden during 2017, and (3) stay at a residency during an MMRP. To obtain a wide range of experiences of MMRPs, theoretical sampling^16^ was used with variation according to age, gender, and rehabilitation clinic. Two deviant cases who had not lived at a residency were also selected to test the emerging theories.^17^ Patients fulfilling the eligibility criteria were initially contacted and informed about the study by the team professionals at the rehabilitation clinics. Patients who had indicated interest in participating in the study were then contacted by the first author (L.S.), who provided further information about the study. An information letter providing comprehensive details about the study, together with a request for consent to participate, was sent to the participants before the interview. All interviewees gave written informed consent prior to the interviews. The final sample comprised 12 people (eight women and four men), aged 20 to 63 years. For more details, see Table 1. Three people who had initially accepted participation in the study dropped out, two because of lack of energy and one who did not give any reason. The included participants had no relationship to the first author (L.S.), who conducted the interviews.

**Table 1.** Characteristic of the participants

	Female or male	Age (years)	Pain duration (years)	Livelihood	Married or cohabitating (children at home, *n*)	Stay in the residency
1	F	57	3	SA 100%	Yes (0)	Yes
2	F	47	15	SA 100%	No (1)	Yes
3	F	43	8	SA 100%	Yes (3)	Yes
4	M	20	5	JS 100%	No^a^	Yes^b^
5	F	33	18	JS 100%	Yes (2)	Yes
6	F	47	27	SA 50%, JS 50%	No (0)	Yes
7	F	63	8	Work 50%, SA 50%	No (0)	Yes^b^
8	F	45	18	SA 100%	Yes (2)	Yes
9	F	24	14	Work 50%, JS 50%	No (0)	No
10	M	42	14	Work 75%, 25% SA	No (0)	No
11	M	34	11	JS 100%	Yes (2)	Yes
12	M	46	2	Work 100%	Yes (2)	Yes

^a^Living with parents.

^b^Stayed the first week at home and with relatives.

F = female; M = male; SA = sick absence; JS = job seeker.

### Data Collection

In accordance with the participants’ wishes, eight participants were interviewed in their homes, three at the rehabilitation clinic, and one at work. A semistructured interview guide with open-ended questions covered various aspects of the participants’ experiences of living at a residency during an MMRP. The interviews started with an open-ended question: “Can you describe your experience of participating in an MMRP?” and was followed up with questions about their experiences of staying at a residency as well as their thoughts and feelings before, during, and after the MMRP. The interviewer developed questions during the interview based on participants’ answers in accordance with an emergent design.^16^ The length of each interview varied between 54 and 72 min. All interviews were individual and were conducted face to face. Interviews were carried out 1 to 6 months after completion the MMRP. All interviews were audio-recorded and transcribed verbatim by the first author.

### Analysis

**Figure 1.**
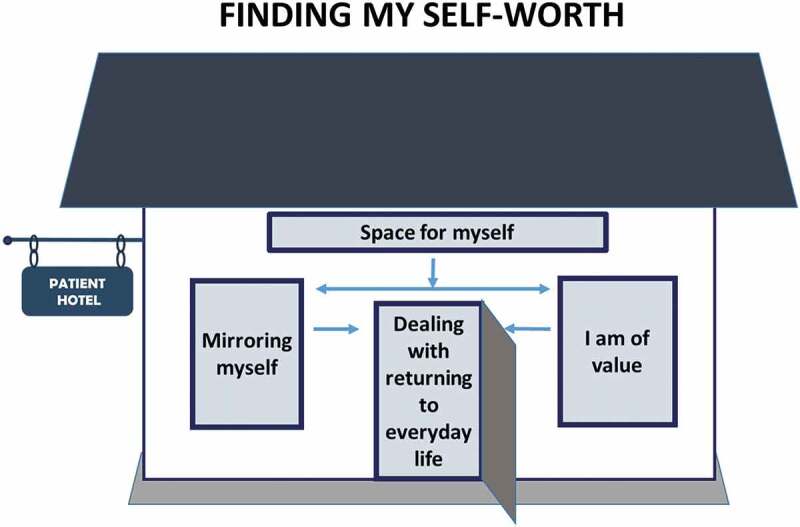
The theoretical model of the process “finding my self-worth” while living in the residency during the MMRP was facilitated by the four categories: (1) space for myself; (2) mirroring myself; (3) I am of value; and (4) dealing with returning to everyday life

An emergent design was used in which the interview and analysis continued and developed in parallel. The original Swedish transcripts were used for the coding process. The analysis was carried out in two steps: firstly, the coding and categorizing of the interviews and, secondly, development of the model. The open coding process was performed by the first author (L.S.) together with two of the other authors (G.S., A.C.K.). The preliminary codes were discussed by the three authors until agreement was reached. In order to increase trustworthiness, all four authors were involved in the formation of categories, subcategories, and the model. The analysis was scrutinized and negotiated among the authors, who met regularly to discuss the emerging results. The findings were presented and discussed at seminars with other researchers in occupational therapy and pain research. Open Code 4.03 software^18^ was used for coding and abstractions. Focus coding identified initial codes that were most significant and that made most analytic sense when categorizing the data.^16^ Codes were sorted into subcategories and categories to find patterns of processes and relations and to identify an overarching core category (Table 2). Axial coding was used to identify subcategories and categories and the links between them.^16^ Theoretical coding^16^ explored and illustrated how the categories and subcategories were related to each other on a more theoretical level. Memos were written after each interview and also during the coding process. The memos were an important part of the analysis and in the development of new categories.^16^ The final findings were presented as a model (Figure 1) that illustrates the process of described experiences when living at a residency. No additional essential information was discovered beyond the eight interviews and thus saturation was considered to have been reached at this point. Furthermore, the content of the additional four interviews confirmed findings from the first eight interviews.

**Table 2.** Subcategories and examples of open codes in the category “Space for myself”

Categories	Subcategories	Codes
Space for myself	Time to focus on myself Time for reflection	Time alone Don’t have to consider other people’s needs
	A break from everyday life	No household chores Preparations prior to being away from home

### Ethics

The studies were approved by the Regional Ethical Review Board in Umea, Medical Faculty of Umea University (Dnr 2015/240-31).

## Results

Analysis of the participants’ experiences of MMRP while living at a residency formed the core category “finding my self-worth,” which permeated all material. In addition to the core category, the model consisted of four categories: “space for myself,” “mirroring myself,” “I am of value,” and “dealing with returning to everyday life,” with interrelated subcategories (Table 3).

**Table 3.** Overview of the core category, categories, and subcategories

Core category	Categories	Subcategories
Finding my self-worth	Space for myself	A break from everyday life Time for reflection
	Mirroring myself	I’m not alone Looking back and reflecting on the old me
	I am of value	Taking care of myself Being true to myself when interacting with others
	Dealing with returning to everyday life	Acquiring new everyday habits Support from others to maintain new routines

The analyses also resulted in a model illustrating the process of finding one’s self-worth (Figure 1). The residency provided a place where participants with chronic pain were able to have time for themselves both physically (time to be alone) and mentally (time to think). This gave them an opportunity to mirror themselves in interactions with others. When reflecting together, the participants gained insights about their own self-worth and found that they were of value. This was significant after the rehabilitation when the participants had to deal with returning to everyday life. A presentation of the categories is given below. Representative quotations are provided.

### Space for Myself

Most of the participants had many obligations in their everyday lives at home. They had struggled to live up to their own and other people’s expectations. Participating in an MMRP and living at a residency was an opportunity for them to have space for themselves, be relieved of responsibilities at home, and to reflect and see to their own needs.

Participants described how living at a residency during the MMRP forced them to spend time without their families. The first days at the residency caused them stress because of feelings of restlessness due to not being involved in the everyday activities they were used to performing at home. After a few days they described a feeling of inner calm. The stay at the residency provided an opportunity for them to reflect on their situation and participants were able to focus on their rehabilitation.

Because I was staying at the hotel, I was ”forced” to be there. For me, this [staying at the hotel] was really good; my whole focus was on myself and I didn’t need to think about anything else. I was there in order to focus on learning about myself. (I 3)

In addition, living at the residency contributed to an awareness of the participants’ habits and roles at home, some of which were undertaken voluntarily, whereas others were forced upon them. They also reflected that their stay at the residency contributed to healthier eating habits such as regular meals and eating more vegetables. It was a new experience for them to prioritize their own desires and decide for themselves what they wanted and needed to do; for example, to socialize with others or just spend time alone. Having time for reflection at the residency alone and having the opportunity to reflect with the other participants during the evenings was described as a privilege.

The participants described both positive and negative experiences with regard to living at the residency. For example, leaving their homes in order to participate in the MMRP was described as an opportunity to have a break from the obligations of everyday life. However, some participants expressed feelings of anxiety about being away from family and not being in control, whereas others were confident that their families would cope with their everyday lives themselves.

All women with young children expressed that they had made preparations at home before leaving for the MMRP by doing household chores and writing to-do lists for their spouses. Living at the residency entailed being apart from close relatives during the MMRP. Living a long distance apart was described as a “useful new experience for the whole family” in practical ways, but the feeling of missing and longing for them was also expressed. Some participants who had partners with poor health and children at home described feelings of guilt due to leaving their partner alone with all of the daily chores. Participants who had an equal distribution of responsibility at home were more comfortable about being away from home. The participants described that support from family and friends made it easier to be away from home during the MMRP. Participants who did not live at the residency disclosed only making preparations at work before leaving for the MMRP.

### Mirroring Myself

The category “mirroring myself” described how valuable the participants perceived it was to have the opportunity to meet other people in similar situations and reflect with others about their own behaviors. Becoming aware of their own needs and necessary changes to achieve new goals became a possibility for recovery.

Extra time and space for the participants contributed to interaction with others. The analysis revealed that the participants who lived at the residency during the MMRP spent a large amount of time together with the other patients during the day and in their spare time. The participants described that they performed activities together with the others and inspired each other to try to be involved in new activities in their spare time. The participants felt support from the other patients in the group when they shared common experiences and struggles with the same problems. They expressed that it was a relief to realize they were not alone.

These feelings you get when the pain is really bad and you are exhausted, you never feel rested, you know, like anxiety and you feel bad mentally. … I hadn’t expected that all the others had also felt like that. (I 5)

The stay at the residency gave participants a strong feeling of community. This feeling gave them the courage to share their experiences, feelings, and thoughts. They also encouraged and gave each other hope, strength, and motivation as well as challenged each other to change their behavior by acting or thinking in new ways. If they performed certain activities during the evenings, they could remind each other to use the new knowledge in ergonomics and activity performance.

The participants emphasized the positive aspects of learning from each other. They gave each other new perspectives and opportunities to look back and reflect on their own behaviors. The participants described that they became aware of and reflected on old nonfunctional strategies like the negative pattern with periods of forced activity that led to periods of inactivity due to heightened symptoms. One participant expressed it as follows:

[I used to think like this] you focus too much on whether you have any pain or whether you’re just imagining things. You don’t try hard enough, but now I know that it’s the other way round. I’m not pretending to be ill, I’ve been pretending to be healthy. (I 6)

The participants reflected that the consequences of their pain led to limited ability and energy to engage in meaningful activities. This, together with their increased sensitivity to stimulation, made it difficult to participate in demanding social contexts and led to avoidance of activities they usually enjoyed being involved in.

I wanted to have better control over the pain so I avoided my family and friends; I realized that if I continued like that then … I would lose my family and friends. (I 4)

When the participants looked back together and reflected on how their lives had changes, they realized that they did not want to live as they did before the MMRP. Those insights provided motivation and inspiration to change their behaviors and try to achieve their new individual goals.

However, some negative experiences were also expressed by the participants. One of the participants described difficulties in relating to the other group members because of the different life situations at the time of MMRP; that is, different ages and different family and work situations.

### I Am of Value

The participants described that the insights they gained when having space for themselves and the opportunity to “mirror themselves” had contributed to them becoming aware of their own value. They had started to take responsibility for themselves and their own choices.

The participants emphasized that they had improved self-confidence after living at the residency while participating in the MMRP. Mirroring themselves in other patients, especially at the residency, led to increased confidence and meant they could start to focus on themselves, take responsibility for their lives, and prioritize being involved in meaningful activities. Participants also described how they now allowed themselves to have days with less activities and did not feel guilty about doing so.

Just because my body feels better one day, I’m not going to exert myself and overdo things that day in order to try to catch up and do all the things that I was not able to do when I was incapacitated because of pain. (I 7)

The participants described a more comfortable feeling of harmony after the MMRP. The participants reported that the opportunity to reflect, discuss, and practice new strategies while being away from home, living at the residency, had contributed to the sense of harmony at home. They described that they now used strategies to live “in the moment” and behave differently when doing activities; that is, they allow themselves to take breaks, rest at work, and perform meaningful activities despite having pain.

I have changed my focus in order to find a balance between what I think is worth doing and not doing, for example, going to the cinema with some friends is worth the pain and then I can put up with it [the pain] since I get so much pleasure from the activity. (I 4)

Participants’ insights regarding their own value improved their courage in asking for support and allowing other people to help them to a greater extent. One participant reflected on that during the interview:

I ask for help without thinking that I should have done the task myself. I’ve never done that before; my motto has been that a capable woman manages everything herself. (I 5)

The participants described that they had acquired an awareness of their own needs and limitations. This awareness contributed to differentiation in relation to work and everyday tasks. They described that they valued their work effort more than before MMRP, as described in this quotation:

When I increased my working hours to 6 hours and felt that it wasn’t working, I dared to say that we must go back to 4 hours. I would never have done that before. I would have kept going until my body packed up completely and then I would have been 100% off work again. I refuse to do that anymore! (I 2)

After the MMRP, the participants described how they felt affirmed and were given the courage to formulate their problems in a new way. This enabled them to communicate differently with relatives, friends, managers, and coworkers, which increased other people’s understanding of their situation.

The analysis revealed that participants who lived at the residency had great support from each other in the process of valuing themselves. This was particularly evident in the evenings, after the sessions, where they discussed and also practiced new behaviors. The participants who did not live at the residency also described that they changed their way of valuing themselves but did not talk about it to the same extent as those who stayed at the residency.

### Dealing with Returning to Everyday Life

During the MMRP and the evenings, at the residency, the participants shared their thoughts about how they should achieve their individual goals and their feelings regarding preparing for their return to life at home. Despite this, they described the process of returning to everyday life with new knowledge, experiences, and a willingness to make changes as being difficult. Participants were aware that if they were to bring about real changes in their lives, it had to be done in the home environment.

The participants who lived at a residency during the MMRP had the opportunity to return home during the weekends. Some of them perceived this as demanding. They described the weekends or weeks between MMRP sessions at home as being hectic and with no time for reflection or recovery. Several of the participants said that their intention had been to practice their acquired knowledge at home but that integrating their new knowledge with their previous roles and routines could be challenging. Participants who had children described how they felt they had to make up for the time that they had been away from their families.

I was supposed to include everything I had learnt there and put it into practice at home and it just doesn’t really work like that, and, well, I panicked, include this and think about that and then on top of that, be with the family. I felt panic. (I 7)

However, after a while, going back home was seen as a good opportunity to put the knowledge acquired from MMRP into practice. Back at the rehabilitation clinic they were able to share their experiences with the other participants in the group, which was described as an important element of the rehabilitation.

The participants described how demanding it was to apply the new strategies consistently and how easy it was to fall back into old routines when they returned to normal life. In addition, they said it was difficult to focus on their own needs when they were together with their families. The analysis showed that participants who lived alone or who did not have their family with them found it easier to apply the new strategies.

Male participants found it easier to change their behavior at their workplaces and had better support from employers compared with women who had a job. From the women’s point of view, it was difficult to incorporate their new knowledge at work because their managers were not able to adapt any of their work tasks.

Participants also said that support from their family, friends, and managers was essential for them to maintain and develop the tools they had acquired during the MMRP in order to achieve individual goals. The participants described that after the MMRP they felt that their family members and managers at work were more understanding, but at the same time they experienced some incomprehension through remarks such as “Aren’t you cured now that you’ve been to rehab?” Some of the participants also said that relatives became overly anxious and were afraid that the pain would get worse, which created frustration.

Additionally, participants were surprised that they had been able to perform so many changes in their lives in such a short time. They described how they had a new way of thinking and acting and that their behavior had changed, whereas their families were the same as before. This became very clear for some of the participants, who said that their partners had not kept up with the changes in the same way.

Several of the participants also described how they were encouraged by other group members via social media after the MMRP to continue the changes they had made in their lives. All participants expressed a desire to meet the other participants in a face-to-face reunion to exchange experiences and replenish new knowledge in order to develop and maintain their new ways of acting and keep up their new strategies.

## Discussion

The results of this study illustrate the process of restoring value when patients with chronic pain participate in an MMRP while living in a residency far from home. Participants described having space for themselves, mirroring themselves, and realizing their own value as a person. This represents steps in the process of regaining the feeling of being an important person, which prepared the participants for dealing with returning to their everyday lives. Our research has demonstrated the process of finding self-worth as a person and the benefits and challenges of living at a residency. As far as we know, no other study has focused on the role of staying at a residency during participation in an MMRP for patients with chronic pain.

Our findings suggest that it is not only important to participate in an MMRP but it is also essential to consider the context and where the rehabilitation takes place. Our study showed that it was important for participants to live at a residency in order to have space for themselves to find their own self-worth and an opportunity to live a meaningful life. This is in line with a study by Lilliehorn and Salander,^15^ who noted how patients who stayed at a residency while being treated for breast cancer could focus on themselves due to having more free time and did not have to take other people’s interests into account.

The opportunity to reflect on individual and contextual factors with others in the same situation was perceived by our participants as being essential for understanding one’s self-worth. The fact that they were living at a residency together with other people in similar situations and were able to share their experiences in a free and open atmosphere meant that the group members built up special relationships with each other. This has been reported earlier in a study of patients who lived at a residency during treatments such as radiotherapy.^15^

Our participants expressed that meeting others in the same situation created opportunities for them to mirror themselves and gave them new perspectives regarding themselves. To stay in a context with other people in the same situation can be perceived as valuable and helpful for the rehabilitation process. This has been observed in several studies of participants with chronic pain.^10,15,19–22^

A turning point for most of the participants in our study was the opportunity for reflection and to mirror themselves. Participants became aware of their own strengths and aware that they could influence their own situation and that they were responsible for changing their situation. This also empowered participants to take an active role in the rehabilitation process, which has also been reported in a previous study of patients with chronic pain.^11,20^ According to Strauss,^23^ a turning point is the point in an individual’s life where he or she needs to reevaluate, revise, re-see, and re-judge his or her situation. Being exposed to major changes implicates a risk of losing one’s self and raises questions such as “Who am I really?” This generates an individual’s need to find and challenge his or her own identity.^23^

Our findings support the assumptions that an MMRP offered to people with chronic pain is a process to find strategies to make it possible to maintain a meaningful life despite pain. This is in accordance with a recently published study by Lennox Thompson et al.,^24^ who described a process of living well with chronic pain. One phase in this process is to decide to “get on with life as it is now” by pursuing meaningful occupations instead of seeking control of pain.

The new knowledge acquired by our participants through MMRP and discussions with other patients in similar situations gave rise to increased self-esteem and insight into the participants’ own importance and self-worth. Hållstam et al.^20^ described the value of a sense of personal significance and how, by helping others, people can strengthen their own self-esteem. Receiving confirmation from others and the realization of being a credible person increases self-esteem, provides strength, and gives people the confidence to listen to themselves and thereby identify what is valuable to them. This is in line with a study by Steihaug,^25^ who pointed out that by negotiating with oneself and others, participants with chronic muscle pain gain insight into what they want and can express and interpret their experiences. In addition, Strauss^23^ described that communication and interaction with others and sharing future and past judgments and experiences contributes to the development of individuals and enables them to see themselves from a new perspective.

Though living at a residency during rehabilitation was useful for our participants, the real changes in their lives had to be made after they returned to their home environment. Using new strategies was perceived by the participants as challenging, and this affected not only themselves but also their social environment, such as family and friends. It has been shown in another study of patients with chronic pain^23,26^ that family members, friends, and colleagues play an important role in helping to maintain new strategies. Research has also shown that when patients learn and understand what aspects of their life are important to them, it is easier for them to be true to themselves and to others.^24^

An interesting finding in our study was that men reported that it was easier to incorporate their new knowledge at work and to get support from their employers than women did. Côté and Coutu^27^ found that women experienced disbelief more often than men did. It is important that employers are aware of this in the future and support persons with chronic pain at their workplaces during and after rehabilitation. Several studies have reported positive effects of MMRPs,^4,6,7^ yet still little is known about how patients experience participation in an MMRP in specialist care clinics in northern Sweden, which is often combined with a stay at a residency. The findings of this study indicate that living at a residency while participating in an MMRP can be perceived as an advantage that further enables participants to focus on their own rehabilitation compared to living at home. The MMRP was an outpatient program and the participants were staying at a designated hotel mainly because of geographical reasons. However, some of the findings can probably be transferred to other outpatient situations because participants who did not stay at the residency also described that they changed their way of valuing themselves. They also described that reflections about their thoughts and behavior during the sessions with the other patients were valuable. Most MMRPs are conducted in group formats, and studies often focus on the content of the programs. The results of our study indicate the importance of also considering the interaction between the patients participating in the MMRP.

## Methodological Considerations

The main strength of our study is the patient perspective, consisting of participants’ experiences of living at a residency during an MMRP. In total, 12 participants from two different clinics provided a broad perspective of the investigated topic. Data were collected 1 to 6 months after the MMRP and the participants were looking back from that point of time and thus had had time for reflection at home. One limitation is the risk that only patients with positive experiences of MMRP participated in the study. However, our results showed that the data involved both positive and negative experiences of participating in an MMRP.

The grounded theory method supported the researchers to remain focused on what the participants expressed and to understand the participants’ perspectives and processes. To ensure trustworthiness, several actions were taken during data collection and analysis. First, a process of triangulation^17^ was used, where the researcher involved had different competencies and perspectives. The authors’ different professional backgrounds (occupational therapist, physician, and physiotherapist) contributed a wide range of skills and experience of pain rehabilitation to the study. The interviews were performed by the first author (L.S.). The author’s pre-understanding was discussed continuously to ensure neutrality.^17^

In addition, professional experiences can enhance sensitivity, which means being able to present the participants’ views through engagement in the data. Peer debriefing^17^ was used at seminars in the research unit where the preliminary findings were presented and discussed in order to further enrich the data analysis.
